# Low serum free triiodothyronineis level predicts worse outcome of patients with severe fever with thrombocytopenia syndrome

**DOI:** 10.1186/s12879-022-07367-6

**Published:** 2022-04-20

**Authors:** Li Wang, Youde Liu, Haifeng Yu, Kun Ding, Zhiqiang Zou

**Affiliations:** Infectious Disease Department, Qishan Hospital of Yantai, 62 Huanshan Road, Zhifu District, Yantai, 264001 Shandong The People’s Republic of China

**Keywords:** Severe fever with thrombocytopenia syndrome, Thyroid function, Nervous symptom, Outcome

## Abstract

**Background:**

Severe fever with thrombocytopenia syndrome (SFTS) caused by phlebovirus results in neuropsychiatric symptoms, multiorgan dysfunction and significant mortality. We aimed to evaluate the thyroid function in SFTS patients, elucidate its association with neuropsychiatric manifestations, disease severity, and prognosis, retrospectively.

**Methods:**

Serum levels of free triiodothyronine (FT3), free thyroxine (FT4) and thyroid stimulating hormone (TSH) were compared between survivors and non-survivors, between those with and without nervous symptoms at baseline, and at baseline and remission. Logistic regression analysis was utilized to determine independent risk factors for mortality. A risk model based on risk factors was constructed and its prognostic value was evaluated by receiver operating characteristic (ROC) curve.

**Results:**

A total of 207 SFTS cases with thyroid function data enrolled from January 2016 to January 2020 were included with 34 patients (16.4%) died. Baseline serum levels of FT3, TSH (*p* < 0.001), and FT3/FT4 ratio (*p* < 0.05) were significantly decreased in nonsurvivors than in survivors. Prevalence of low serum FT3 in nonsurvivors (81.8%) was greater than in survivors (41.3%). FT3 level (*p* < 0.001) was markedly reduced in patients with central neurological symptoms than those without. FT3 and FT4 levels were increased in remission than at baseline (*p* < 0.001). Logistic regression analysis showed that age (OR 0.92, 95% CI 0.868–0.958) and serum FT3 level (OR 3.055, 95% CI 1.494–6.248) were the independent risk factors for mortality. A risk model based on age and FT3 had a high predictive value for mortality (AUC = 0.818, 95% CI 0.795–0.868) at a cutoff value of > 3.39.

**Conclusions:**

Low serum FT3 level was associated with a worse outcome of SFTS patients.

## Background

Severe fever with thrombocytopenia syndrome (SFTS) is an emerging virus infectious disease caused by SFTS virus (SFTSV), a novel tick-borne bunyavirus of the genus Phlebovirus and family Phenuiviridae. SFTS has been reported endemic in some of Eastern Asia and Southeast Asia countries, including China, Korea, and Japan, which has a high mortality rate in humans [1]. Clinical symptoms of SFTS involve multi-organ dysfunction manifestations. Central nervous system (CNS) injury presented as acute encephalopathy/encephalitis symptoms are a severe and common complication of SFTS, mainly including headache, confusion and seizure [2]. It has been reported that approximately 19% of SFTS patients developed encephalitis and fatal outcome occurred in 44.7% of these patients [3].

The exact mechanisms of SFTS-associated encephalopathy/encephalitis (SFTSAE) were unclear. Several factors have been suggested to be associated with pathogenesis of SFTSAE, including direct invasion of SFTSV in the CNS [4] and proinflammatory cytokine storm induced inflammatory response in the CNS [5]. While other manifestations, such as apathy, slurred speech, and limb tremors can not be fully explained by SFTSAE. It has been proposed that SFTSV infection triggered cytokine storm highly resembles the immune activation seen in sepsis [6], and sepsis is often accompanied by thyroid dysfunction called "low triiodothyronine (T3) syndrome" which greatly affects the prognosis of patients [7]. Furthermore, development of peripheral and central neuropathy was observed at the early stage of disease in patients with hypothyroidism [8]. Decreased conduction velocity and sensory function of the tibial and peroneal nerves observed in hypothyroid patients were also present in SFTS patients which might account for neuro-muscular symptoms [9].

Whereas the prevalence of thyroid dysfunction and low T3 syndrome in SFTS has not yet been evaluated. In this study, we intended to assess the incidence of low T3 syndrome in SFTS and its association with neuropsychiatric manifestations, severity of the disease and mortality.

## Patients and methods

### Patients diagnosis and symptom evaluation

Two hundred and seven SFTS patients admitted to our hospital from January 2016 to December 2020 who have thyroid function test results were included. Diagnosis of SFTS was confirmed by detected positive SFTSV from peripheral blood samples using reverse transcription–polymerase chain reaction (RT–PCR). Demographic and physiological parameter including age, gender, body temperature, pulse, respiratory rate and blood pressure were collected at admission. Serum free T3 (FT3), free thyroxine (FT4) and thyroid stimulating hormone (TSH) were detected using an automatic chemiluminescence immunoanalyzer (Mindray CL-6000i). Timing points of estimation of FT3, FT4 and TSH were selected on admission and remission for survivors and only on admission for nosurvivors due to their short hospital stays. Serum biochemical parameters were assayed by Beckman AU5800 automatic biochemical analyzer.

Central neuropsychiatric symptoms and peripheral motor nerve symptoms were evaluated by careful physical examinations. Patients were divided into different groups according to survival state, or whether with or without central neuropsychiatric symptoms and peripheral motor nerve symptoms. Thirty old individuals with no significant cardiovascular disease, endocrine diseases and SFTSV infection who underwent health physical examination were included as controls.

This study was performed according to the Helsinki II Declaration and was approved by the ethics committee of Qishan (Infectious Disease) hospital of Yantai, Shandong, China (Ethics number 202201).

### Statistical analysis

Data are presented as mean ± SD or median (Q_25_–Q_75_). The student *t* test or paired *t* test, Mann–Whitney *U* test or paired nonparametric test and one-way ANOVA were used for the comparison of variables between or among groups. Proportions of variables between groups were compared using χ ^2^ tests. Correlation analysis was done using Spearman's correlation analysis. Multivariate and univariate logistic regression analysis were used to obtain independent risk factors associated with mortality. A risk model based on these independent risk factors was established. Area under the receiver operating characteristic curve (ROC) (AUC) with the highest Youden index was used to evaluate the prognostic values of these factors and the model. The KaplanMeier survival analysis was utilized to compare the cumulative risk for mortality using the log-rank test. SPSS software (version 23.0, IBM, Armonk, NY, USA) and MedCalc software were used for statistical analysis and *p* values < 0.05 were considered significant.

## Results

### Comparison of demographics and baseline biochemical and thyroid function parameters between survivors, nonsurvivors and normal controls

**Fig. 1:**
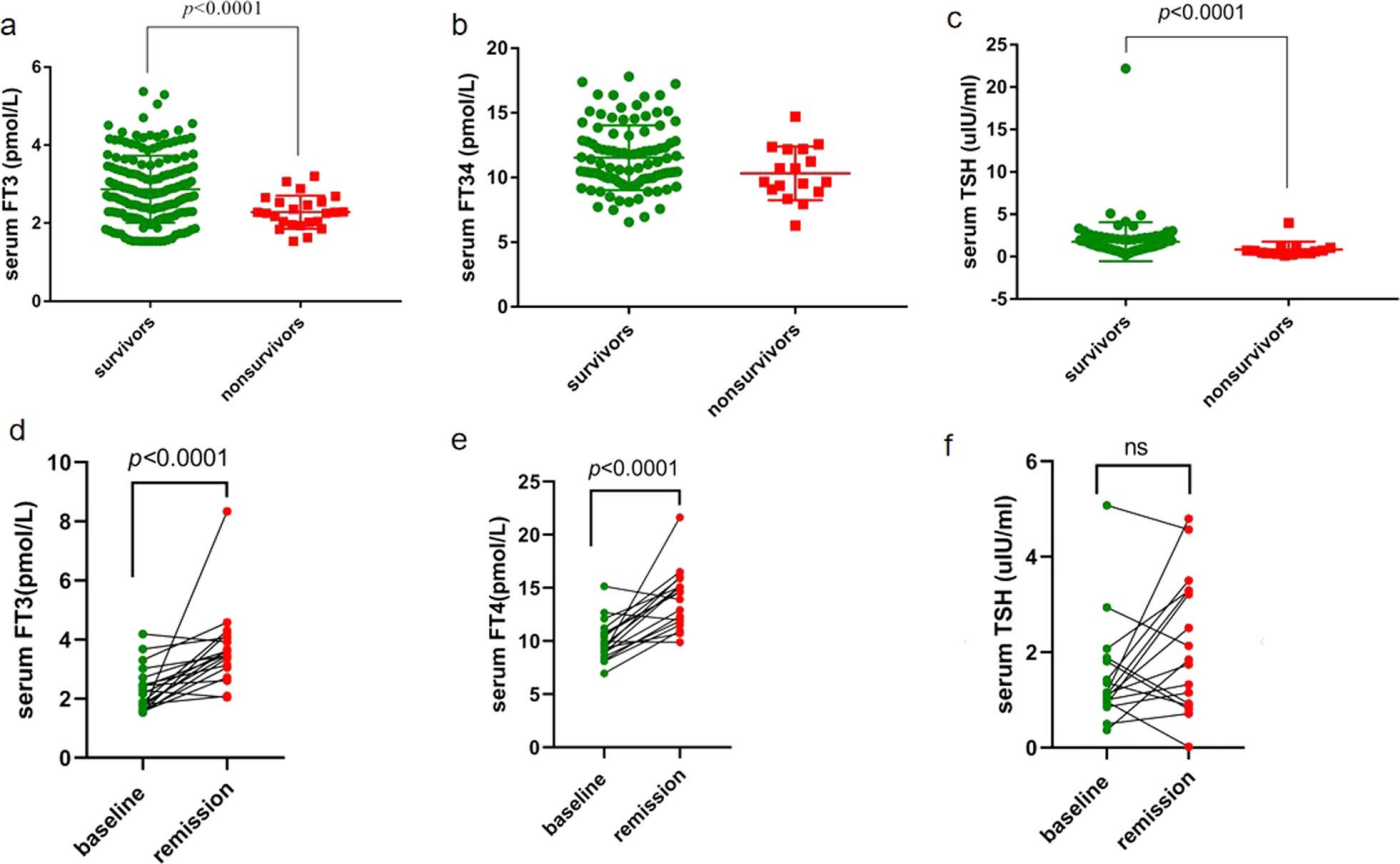
Comparison of serum levels of FT3, FT4 and TSH between survivors and nonsurvivors, and between baseline and remission. **a**–**c** Serum levels of FT3, FT4 and TSH of survivors and nonsurvivors, respectively. **d**–**f** Serum levels of FT3, FT4 and TSH at baseline and remission, respectively

Of 207 SFTS patients, 34 (16.4%) died. Mean age of non-survivors (71.4 ± 10.8 years) was greater than survivors (60.9 ± 10.9 years) (*p* < 0.0001). Percentage of male patients in nonsurvivors (73.5%) was greater than in survivors (43.6%) (*p* < 0.05). Serum levels of FT3 and TSH and FT3/FT4 ratio were decreased dramatically in nonsurvivors compared with those in survivors. Serum levels of FT3 and FT4 and FT3/FT4 ratio were reduced profoundly in survivors and non-survivors compared with those in controls. Prevalence of low serum FT3 in survivors, nonsurvivors and controls were 41.3%, 81.8% and 6.7%, respectively (*p* < 0.0001). Though TSH level has no significant difference between survivors and healthy controls, it is remarkably lower in nonsurvivors lower than in controls. Incidence of nervous symptoms Serum level of Hs-CRP and pulse number were greater in non-survivors than in survivors. Incidence of neurological symptoms (%) for survivors and nonsurvivors were 36.4% and 88.2%, respectively (*p* < 0.0001). Data are presented in Table 1 and Fig. 1a–c.

**Table 1:** Comparison of baseline FT3, FT4, FT3/FT4 ratio, TSH, biochemical indicators and physical parameters in survivors and nonsurvivors [mean ± SD or median (Q_25_–Q_75_)]

Parameters	Survivors	nonsurvivors	controls	*p value
N (%)	173 (83.6)	34 (16.4)	30	–
Age (year)	60.9 ± 10.9^##^	71.4 ± 10.8^###^	55.1 ± 8.1	< 0.0001
M/F (%)	84/89(48.6/51.4)	25/9 (73.5/26.5)	13/17(43.3/56.7)	0.013
FT3 (pmol/L)	2.78 (2.25, 3.44)^###^	2.24 ± 0.42^###^	4.40 ± 0.83	< 0.0001
Prevalence of low serum FT3 (%)	71/172 (41.3)^###^	27/33(81.8)^###^	2/30(6. 7)	< 0.0001
FT4 (pmol/L)	11.05 (9.93, 11.15)^###^ (n = 100)	10.87 ± 2.08^###^ (n = 26)	14.23 ± 2.01	0.37
FT3/FT4 ratio	0.25 ± 0.06^###^ (n = 100)	0.22 ± 0.06^###^ (n = 26)	0.32 ± 0.07	0.016
TSH ( uIU/ml)	1.37 (0.89, 1.96) (n = 98)	0.61 (0.41, 1.06)^##^ (n = 24)	1.11 (0.86,1.72)	< 0.0001
TCH (mmol/L)	3.5 (3.0, 4.18)	3.2 (2.6, 3.9)	–	0.084
Hs-CRP (mmol/L)	5 (1.8,13.3)	9 (5.3,41.0)	–	0.008
Pulse (beats/min)	74 (67,82)	84.0 ± 11.6	–	< 0.0001
Baseline body tempreture (℃)	37.2 (36.3, 38.3)	37.5 (36.7, 38.5)	–	0.233
Highest body tempreture (℃)	38.9 (38.5, 39)	39 (38.5,39.2)	–	0.42
Ctnl (pg/ml)	0.41(0.03, 33.5)	23.9(0.25, 214.6)	–	0.011
CK(U/L)	473 (178.9, 1027.4)	1199.5(485.3, 3523.5)		0.001
CK-MB(U/L)	33.7(21.3, 48.6)	59.1(31.7, 118.5)		0.001
LDH(U/L)	575.0 (386.1, 867.2)	942.2 (636.6, 1302.8)		0.004
HBDH(U/L)	404.3 (280.6, 566.8)	594.6 (438.1, 1114.3)		0.002
Incidence of neurological symptoms (%)	63/173 (36.4)	30/34 (88.2)		< 0.0001

*FT3* free triiodothyronine; *FT4* free tetraiodothyroxine; *TSH* thyroid stimulating hormone; *TCH* total cholesterol; *Hs-CRP* high-sensitivity C-reactive protein; *MAP* mean arterial pressure; *Ctnl* cardiac troponin; *CK* Creatine kinase; *LDH* lactate dehydrogenase; *HBDH* hydroxybutyrate dehydrogenase

*Comparison between survivors and non-survivors

^###^*p* < 0.001, ^##^*p* < 0.01, ^#^*p* < 0.05 in comprison with control

### Comparison of parameters of thyroid function between males and females in survivors and nonsurvivors

Mean age between males and females in survivors and non-survivors had no significant difference. Among thyroid function parameters, only FT3 was decreased markedly in females [2.66 (2.13, 3.36)] in comparison with that in males [2.92 (2.33, 3.50)] in survivors (*p* = 0.033) which suggested that difference of FT3 level between male and female patients had no association with SFTS prognosis. Data are indicated in Table 2.

**Table 2:** Comparison of baseline serum FT3, FT4, FT3/FT4 ratio and TSH between male and female in survivors and nonsurvivors

Parameters	survivors		*p* value	nonsurvivors		*p* value
	male	female		male	female	
Age (year)	63 (51.3,72)	59.4 ± 9.6	0.056	70.1 ± 10.8	74.9 ± 10.8	0.26
	(n = 84)	(n = 89)		(n = 25)	(n = 9)	
FT3 (pmol/L)	2.92 (2.33, 3.50)	2.66 (2.13, 3.36)	**0.033**	2.30 ± 0.36	2.10 ± 0.53	0.23
	(n = 84)	(n = 89)		(n = 25)	(n = 9)	
FT4 (pmol/L)	11.5 ± 2.6	11.6 ± 2.4	0.78	10.8 ± 2.2	11.0 ± 1.9	0.83
	(n = 48)	(n = 50)		(n = 18)	(n = 8)	
FT3/FT4 ratio	0.26 ± 0.06	0.24 ± 0.07	0.14	0.20 (0.19, 0.24)	0.19 ± 0.06	0.2
	(n = 48)	(n = 50)		(n = 18)	(n = 8)	
TSH (uIU/mL)	1.37 (0.89,1.96)	0.61(0.41, 1.06)	0.3	0.68 (0.41, 1.23)	0.56 (0.35, 1.16)	0.83
	(n = 48)	(n = 50)		(n = 16)	(n = 8)	

### Comparison of parameters of thyroid function at baseline and remission in survivors

Serum FT3 and FT4 levels were elevated significantly at remission in comparison with at baseline in the same cohort of patients. Whereas, FT3 level in remission was still markedly lower than controls. FT4 level was comparable with controls in remission. Serum levels of TSH had no significant difference among baseline, remission and controls. Other biochemical parameters, including creatine kinase (CK), CKMB, hydroxybutyrate dehydrogenase (HBDH) and lactate dehydrogenase (LDH) were also decreased dramatically at remission comparing with at baseline. Data are summarized in Table 3 and Fig. 1d–f.

**Table 3:** Comparison of FT3, FT4, FT3/FT4 ratio and TSH at baseline and remission in survivors

	Baseline	Remissiom	Control	*p value
n	19	19	30	–
age	60.9 ± 10.9	60.9 ± 10.6	55.1 ± 8.1	–
M/F (%)	9/10 (47.4/52.6)	9/10 (47.4/52.6)	13/17(43.3/56.7)	–
FT3 (pmol/L)	2.16 (1.6, 2.71)^###^	3.48 (3.05, 4.07)^###^	4.40 ± 0.83	< 0.0001
FT4 (pmol/L)	9.98 ± 1.93^###^	13.91 ± 2.79	14.23 ± 2.01	< 0.0001
FT3/FT4 ratio	0.23 ± 0.08^##^	0.26 ± 0.07	0.98 ± 0.74	0.13
TSH (uIU/mL)	1.15 (0.95, 1.93)	1.84 (0.90, 3.30)	1.11 (0.86,1.72)	0.113
Hs-CRP (mmol/L)	6.3 (1.7, 13.6)	2.3 (1.5, 7.5)	–	0.221
Ctnl(pg/mL)	0.08 (0.02, 0.2)	0.02 (0.006, 0.13)	–	0.403
CK (U/L)	526.4 (147.7, 1208.0)	51.9 (27.7, 96.9)	–	< 0.0001
CK-MB (U/L)	38.7 (23.0, 52.5)	12.0 (9.3, 22.6)	–	< 0.0001
HBDH (U/L)	524.8(338.9, 827.5)	260.9 (210.8, 334.1)	–	< 0.0001
LDH (U/L)	886.7 (485.9, 1528.7)	309.1(261.2, 401.9)	–	< 0.0001

*Ctnl* cardiac troponin; *CK* creatine kinase; *CK-MB* creatine kinase-MB; *HBDH* hydroxybutyrate dehydrogenase; *LDH* lactic dehydrogenase

*Comparison between baseline and remission

^###^*p* < 0.001, ^##^*p* < 0.01 compared with control

**Fig. 2:**
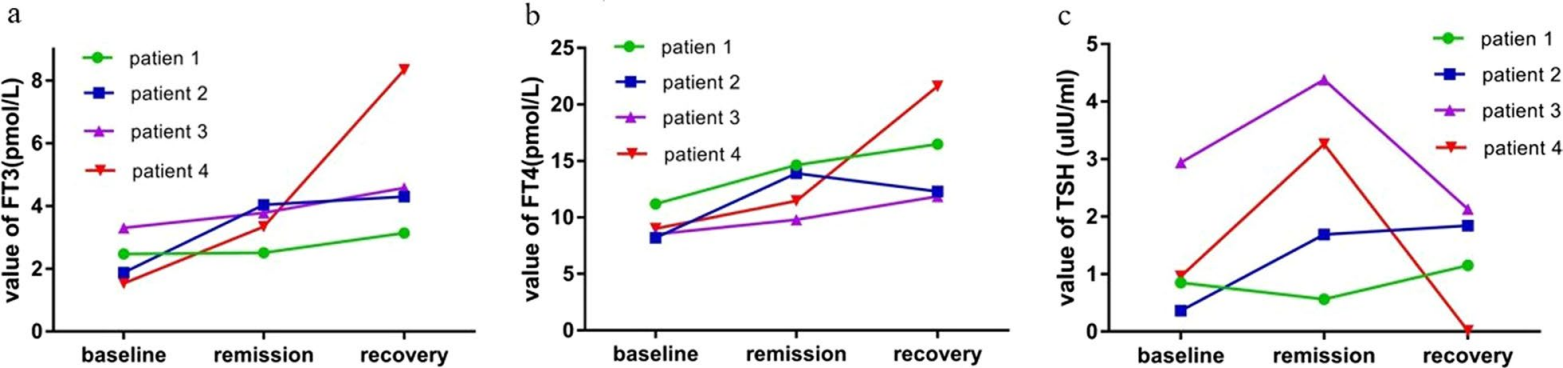
Serum levels of FT3, FT4 and TSH at different time points. **a**–**c** Serum levels of FT3, FT4 and TSH of 4 survivors at baseline, remission and recovery, respectively

A total of 4 patients tested for thyroid function 3 times at admission, remission and recovery, respectively (Fig. 2). Levels of FT3 and FT4 elevated gradually, while TSH level in two of these patients elevated in remission and dropped sharply in recovery.

### Thyroid functions in patients with and without symptoms of central or peripheral nervous system dysfunction in survivors

Mean age of patients with central nervous system symptoms was greater than that of those with peripheral nervous symptoms and without nervous symptoms (*p* < 0.01). Serum FT3 level in patients with central neuropsychiatric symptom was much less than that in those other two groups. Though levels of FT4 and TSH in patients with peripheral and central nervous symptoms were comparable, FT3/FT4 ratio was greater in the former than in the latter (*p* < 0.05). Serum Hs-CRP level has no significant difference among these three groups. CK-MB (*p* < 0.05), HBDH and LDH (*p* < 0.01) were increased profoundly in patients with central nervous symptom. Data are given in Table 4.

**Table 4:** Comparison of baseline FT3, FT4, FT3/FT4 ratio and TSH in patients with and without symptoms of neuropsychiatric dysfunction in survivors

	Without nervous symptom	With central neuropsychiatric symptom	With peripheral neurological symptom
Age (year)	59.2 ± 11.4	65.4 ± 8.9^**^	62.7 ± 9.8
M/F (%)	54/58 (48.2/51.8)	14/18(43.8/56.2)	16/12(57.1/42.9)
FT3 (pmol/L)	2.89 (2.28, 3.53) (n = 111)	2.51 ± 0.75^*^ (n = 31)	2.78 (2.28, 3.58) (n = 28)
Prevalence of low serum FT3 (%)	42/111(37.4)	16/31(51.3)	11/28(39.3)
FT4 (pmol/L)	11.8 ± 2.6 (n = 61)	11.2 ± 2.3 (n = 20)	10.36 (9.73, 13.01) (n = 18)
FT3/FT4 ratio	0.25 ± 0.07 (n = 61)	0.23 ± 0.07 (n = 20)	0.27 ± 0.05^#^ (n = 18)
TSH (uIU/mL)	1.49 (1.00, 2.13) (n = 60)	1.18 (0.74, 1.50) (n = 20)	0.84 (0.60, 4.46) (n = 18)
Baseline body tempreture (℃)	37.5 ± 1.07	37.3 ± 1.90	37.5 ± 1.10
pulse	74.8 ± 11.1	75.8 ± 11.8	72.4 ± 11.4
Hs-CRP (mmol/L)	4.65 (1.80, 13.28)	6.95 (1.73, 18.08)	5.70 (1.80, 17.28)
Ctnl (pg/mL)	0.56 (0.03, 31.2)	0.20 (0.03, 39.1)	14.6 (0.07, 45.4)
CK (U/L)	469.3 (156.8, 952.2)	685.9 (167.5, 1246.3)	526.4 (276.1, 1208.0)
CK-MB (U/L)	30.5 (20.1, 44.8)	41.4 (24.8, 59.5)^*^	40.3(25, 54.2)^*^
HBDH (U/L)	378.6 (264.9, 506.2)	553.9 (337.5, 707.7)^**^	410.2 (300.2, 703.9)
LDH (U/L)	551.7 (355.9, 748.0)	844.2 (478.5, 1225.1)^**^	560.0 (401.1, 1039.7)

*p < 0.05 **p < 0.01 compared with group without nerve symptom; ^#^p < 0.05 compared with group with central neuropsychiatric symptom

### Correlation between serum Hs-CRP and parameters of thyroid function

In survivors, serum FT3 and TSH levels were correlated negatively with serum hs-CRP level at admission, especially FT3 (*p* < 0.0001). In remission, no significant correlation was observed between serum hs-CRP and parameters of thyroid function. While in nonsurvivors, no significant correlation between these parameters.

### Independent risk factor obtaining and risk model construction

Univariate logistic regression analysis showed that age, serum levels of FT3, CK-MB, HBDH and LDH (*p* < 0.2) were independent risk factors for mortality of SFTS patients. Multivariate logistic regression analysis indicated that only age and serum FT3 were the independent risk factors for mortality with odds ratios (OR) (95% CI) were 0.912 (0.868, 0.958) and 3.055 (1.494, 2.658), respectively. Their regression coefficient were 0.093 and − 1.117, respectively. Based on age and serum level of FT3, we constructed a risk score model (M) = 0.093 × age − 1.117 × FT3 for the prediction of mortality (Table 5).

**Table 5:** Predictive values of risk factors and risk model (M) for mortality

	Cutoff values	AUC (95% CI)	SEN (%)	SPE (%)	PPV (%)	NPV (%)	LR+	LR−
Age (year)	> 64	0.752 (0.687, 0.809)	78.3	61.1	21.2	95.5	2.01	0.36
FT3 (pmol/L)	< 2.69	0.727 (0.660, 0.786)	87.0	55.0	20.6	96.9	1.93	0.24
M	> 3.39	0.818 (0.759, 0.868)	82.0	73.0	29.0	96.0	2.6	0.09

*AUC* area under ROC curve; *SEN* sensitivity; *SPE* specificity; *PPV* positive predictive value; *NPV* negative predictive value; *LR*+ positive likelihood ratio; *LR−* negative likelihood ratio; *M* risk score model

### Predictive values of FT3 and the risk model for the prognosis of SFTS patients

**Fig. 3:**
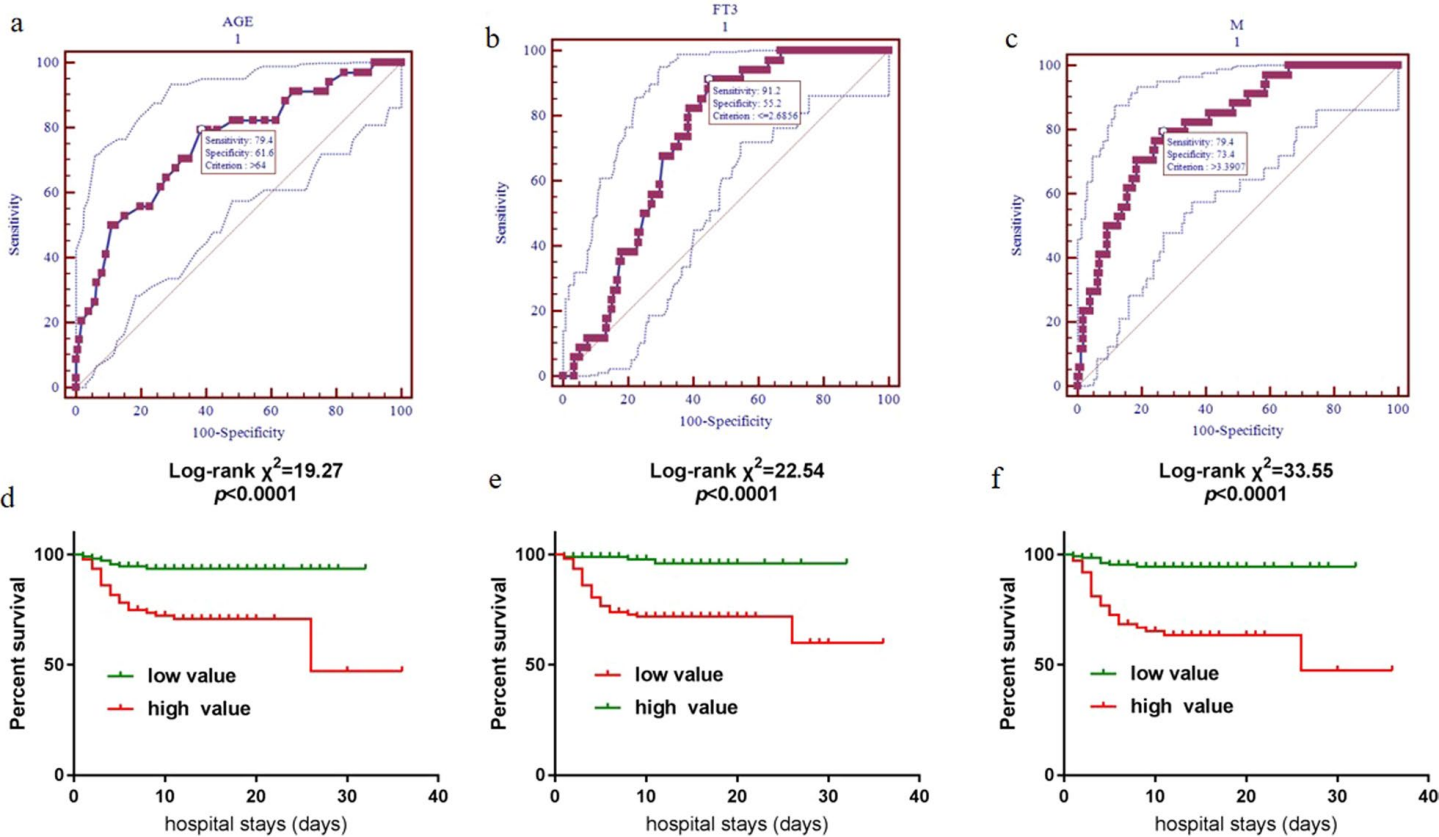
ROC and survival curve of age, serum FT3 and risk model (M) for SFTS prognosis. **a**–**c** ROC curves of age, serum FT3 and risk model for predicting SFTS mortality. **d**-**f** Survival curves of age, serum FT3 and risk model for SFTS prognosis

The cutoff values and AUCs (95% CI) of age, FT3 and risk model (M) with sensitivity (SEN), specificity (SPE), positive predictive value (PPV), negative predictive value (NPV), positive likelihood ratio (LR +) and negative likelihood ratio (LR-) for predicting mortality of SFTS patients obtained by ROC analysis were included in Table 5 and Fig. 3a–c. Log-rank test of survival analysis showed that the overall survival rate had remarkable difference between low and high value groups at the cutoff values of age, FT3 and the model (M) with the highest Youden index (Fig. 3d–f).

## Discussion

In this research, we found that the prevalence of low serum FT3 level was higher in nonsurvivors (81.8%) than in survivors (41.3%) (Table 1) and low serum level of FT3 was the independent risk factor of mortality for SFTS patients (Table 5). Though the prevalence of low serum FT3 was comparable among patients with central neuropsychiatric, peripheral neurological symptoms and those with no neurological symptoms, serum FT3 level was markedly lower in patients with central neuropsychiatric symptom than those in the other two groups (Table 4) which indicated that degree of low FT3 level was associated with central neuropsychiatric symptom.

Consistent with our results, previous studied had demonstrated that development of encephalopathy/encephalitis was associated with worse outcome in SFTS patients [3]. SFTSV genome detected in cerebrospinal fluid (CSF) in SFTS patients suggests that direct invasion of SFTSV in the CNS may play an important role in the pathogenesis of SFTSAE [3]. Our results showed that both of serum FT3 level and TSH level were decreased dramatically in nonsurvivors, which suggested that hormone regulation in hypothalamus-pituitary-thyroid axis was seriously harmed. And SFTSAE may be one of the reasons leading to damage of hypothalamus and pituitary and therefor resulting in hypothyroidism and low serum FT3 level, especially in patients of mortality.

On the other hand, indirect effect of the inflammation and cytokine storm triggered by SFTSV infection also involved in the disturbance of consciousness [3, 4]. Consistent with this, inhibition of systemic inflammation by short-term methylprednisolone pulse therapy resulted in a favorable outcome in some patients. And this remedy led to no long term damage to neurological system [10]. Low FT3 syndrome may also occur in inflammatory response syndrome (SIRS) or sepsis [8, 11]. In line with this, we found that baseline serum FT3, TSH levels and Hs-CRP level were negatively correlated in survivors and no noteworthy correlations were observed between these parameters in remission and in nonsurvivors. These results indicated that systemic inflammatory response might play a key role in hypothalamus-pituitary-thyroid axis dysregulation and low FT3 syndrome development in survivors. Furthermore, though FT3 and FT4 levels were elevated significantly in admission than at baseline, TSH levels were still comparable with those at baseline which suggested that it might need a long period for the normalization of the hypothalamus-pituitary-thyroid axis hormone regulation or it might never return to normal regulation.

Nevertheless, our results indicated that low FT3 syndrome was mainly attributed to CNS injury and central hypothyroidism in SFTS patients, it might also be resulted from thyroid injury itself. Previous studies demonstrated that SFTSV antigens and SFTSV-nucleocapsid protein (NP) were positive in multiple organs including the heart, lung, kidney, spleen and CNS detected by immunohistochemistry and immunoblasts [12, 13] which indicated that SFTSV led to systemic infection. We speculate that thyroid can also be infected and results in hypothyroidism which needs further investigation.

It has been shown that hypothyroidism is more common among older people in comparison to the youngsters. Because the hypothalamic-pituitary-thyroid axis and its secreted hormones undergo significant changes with the physiological aging [14]. Our results showed that age of nonsurvivors was greater than that of survivors, and age was an independent risk factor for mortality, which suggested that preexist hypothyroidism might lead to a worse outcome in old patients. A risk model based on the combination of age and serum FT3 had a high prognostic predictive for adverse outcome of SFTS patients.

Thyroid hormones (THs) play an essential role in both the innate and adaptive immune responses [15]. SFTSV infection induced hypothyroidism can in turn result in defective immune responses and aggravate infection leading to a vicious cycle. Here, we propose a potential treatment strategy supplement with THs and other supportive measures for the protection and promotion of recovery of severe hypothyroidism in SFTS.

There are several limitations in our study. Firstly, few second THs testing data were available due to short hospital stays in nonsurvivors which might affect analysis results. Secondly, lack of tissue infection results including thyroid and brain in nonsurvivors. Thirdly, lack of follow-up data of thyroid functions after being discharged from hospital. Fourthly, no data were available on effects of inflammation inhibition on thyroid function due to insufficient patients received glucocorticoid treatment. Lastly, data of changes of hypothalamus and pituitary hormone levels during SFTS disease course were absent.

## Conclusions

In all, low serum FT3 was usually present in SFTS patients and was associated with an adverse outcome. Serum FT3 level was much lower in patients with central neuropsychiatric symptom than that in patients without nervous manifestations. Age and serum FT3 were the independent risk factors associated with mortality of SFTS patients. The risk score model based on them holds a high predictive value for in-hospital mortality.

## Data Availability

All relevant data are within the paper.

## Abbreviations

AUCs: Area under ROC curve
CNS: Central nervous system
FT3: Free triiodothyronine
FT4: Free thyroxine
hs-CRP: High-sensitivity C-reactive protein
LR+: Positive likelihood ratio
LR−: Negative likelihood ratio
NPV: Negative predictive value
PPV: Positive predictive value
ROC: Receiver-operating characteristic
RT–PCR: Reverse transcription–polymerase chain reaction
SEN: Sensitivity
SPE: Specificity
SFTS: Severe fever with thrombocytopenia syndrome
SFTSV: SFTS virus
TSH: Thyroid stimulating hormone
SFTSAE: SFTS-associated encephalopathy/encephalitis
